# Fully Guided Implant Placement and Immediate Loading for the Restoration of the Edentulous Maxilla with a Fixed Dental Prosthesis: Report of Two Clinical Cases

**DOI:** 10.3390/dj14060373

**Published:** 2026-06-16

**Authors:** Panagiotis Lampropoulos, Nikitas Sykaras, Nikolaos Nikitas Giannakopoulos, Stefanos Kourtis

**Affiliations:** 1 Private Praxis, 17121 Athens, Greece; plampropoulos@dentalimplant.gr; 2 Department of Prosthodontics, Dental School, National and Kapodistrian University of Athens, 11527 Athens, Greece; nsykaras@dent.uoa.gr (N.S.); nikgian@dent.uoa.gr (N.N.G.)

**Keywords:** guided implant placement, immediate loading, fixed dental prosthesis, edentulous maxilla, dental implants, digital planning

## Abstract

**Objective:** The purpose of these two clinical cases reports was to present the variations in the surgical and prosthetic procedures related to an implant-supported fixed dental prosthesis (FDP) in the edentulous maxilla, following a fully guided implantation protocol and immediate loading. **Case presentation**: Two patients, one with terminal dentition and one with a completely edentulous maxilla, expressed the desire for immediate rehabilitation with an FDP and avoidance of multiple and complex surgical procedures. The clinical protocol for both cases included meticulous presurgical planning combining radiographic examination, diagnostic wax-up (conventional or digital), prosthetically driven digital planning of implant placement, fabrication of a surgical guide for fully guided implant installation, and delivery of a screw-retained fixed restoration. There was no report of any implant failure; the FDPs could be delivered as planned, and both patients expressed their satisfaction with the functional and esthetic outcomes. The clinical situation was stable at the recall.

## 1. Introduction

Guided dental implant placement is employed in a wide range of clinical scenarios, from single tooth replacements to complex full arch restorations.

In cases where the patient’s anatomy is challenging, with insufficient bone volume in height or width, or proximity to the maxillary sinus or the inferior alveolar nerve, guided implant placement ensures that the implant is placed in the optimal prosthetic position, avoiding critical anatomical structures. This reduces the risk of complications and improves the long-term stability of the implant and the success of the prosthesis [1]. In situations where an implant is placed immediately after tooth extraction, guided surgery ensures that the implant is correctly positioned in the fresh extraction socket. This is particularly important to maintain proper alignment of the implant with the prosthetic restoration and to preserve the surrounding bone and soft tissues [2].

For patients requiring multiple implants for partial or full arch fixed dental prosthesis (FDP), guided implant placement facilitates correct spacing and ensures that all implants are placed with the ideal angulation to facilitate the design and fabrication of the prosthetic restoration which can follow the immediate or conventional loading protocol [3].

In fully edentulous patients, guided implant placement may direct the precise placement of multiple implants to support a full-arch FDP. Mucosa- or bone-supported guides are used in these cases, allowing accurate placement even in the absence of teeth. The main advantage of guided implantation is the level of precision it offers. This precision reduces the risk of complications such as nerve damage, sinus perforation and implant misalignment [4]. Implantation guided by a physical template reduces the variability associated with freehand implant installation and the possibility of human error, resulting in more predictable results and a lower risk of implant failure [5]. Pre-operative planning with reverse engineering of the final FDP and the use of a surgical guide can often reduce the time required for surgery. Since the indicated optimal position is already assigned for each implant site, the surgeon can execute the plan more efficiently and stress-free for the benefit of both the patient and the surgical team [6].

Guided implant placement can be performed with flapless surgery, which is a less invasive technique associated with reduced trauma and post-operative complications such as inflammation, bleeding, hematoma and swelling, while improving patient acceptance and comfort. It is considered a valuable alternative to traditional mucoperiosteal flap elevation and suturing, as it preserves the osteogenic potential and blood supply of the surgical field [7].

In anterior region cases, where esthetics is a priority, guided implant placement ensures that the implant is placed in the optimal position and angulation to support the final prosthetic restoration. This helps to ensure that the prosthesis design supports the esthetic requirements following the pre-treatment planning and evaluation.

Despite its many benefits, guided implantation has some challenges and limitations that need to be considered. First, the accuracy of implant position is highly dependent on the quality of the initial Cone Beam Computer Tomography (CBCT) scan, and in cases of backward planning, it depends on the accuracy of superimposition of various STL (Standard Tesselation Language) files [8,9].

Moreover, the surgical guides do not allow for deviations that might be necessary to adapt the plan intraoperatively. Another barrier to the widespread adoption of guided implantation is its increased cost. The need for CBCT, dedicated planning software and 3D printed surgical guides adds to the overall cost of the procedure, and it may be redundant in cases where the implant procedure is straightforward [10].

While guided implantation reduces the technical challenges of implant placement, it requires a learning curve for both clinicians and dental technicians. The dentists performing the surgical procedures need to become proficient in the use of digital planning software, and dental technicians need to be skilled at fabricating accurate surgical guides. Based on the above, any errors in digital planning or guide fabrication can lead to suboptimal results during surgery [11].

The aim of this clinical report is to demonstrate current techniques and variations in surgical and prosthetic procedures for two patients with an indication for implant-supported full-arch FDP restorations in the maxilla, following fully guided surgery and digital technology applications, with at least one year of follow-up.

Cases presentation

All clinical data presented in this report were selected and handled according to the Helsinki declaration, and the patients signed an informed consent form permitting the communication of clinical images in scientific forums.

**Case 1**

A 56-year-old male patient in good general health, a non-smoker and under no medication, presented to a private dental clinic for prosthetic rehabilitation. He is a professional woodwind musician with great concern about his anterior teeth because of his profession.

**Figure 1:**
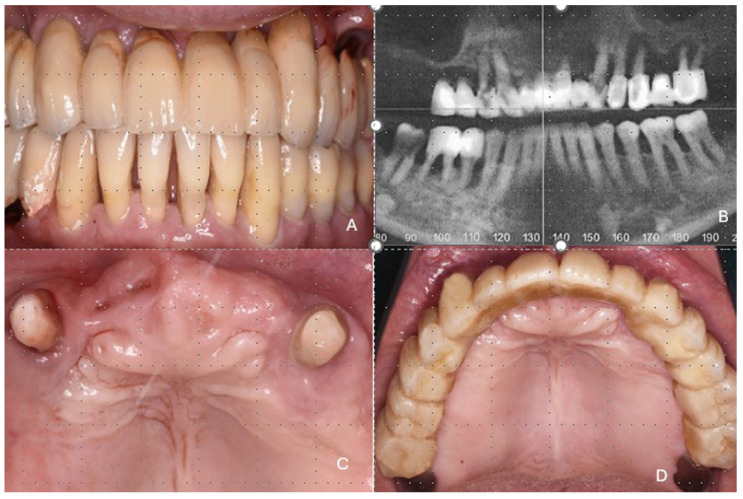
(**A**) Initial clinical situation, (**B**) Initial radiographic examination (from CBCT), (**C**) Clinical situation after extraction of the maxillary teeth except for the canines, (**D**) The tooth- and tissue-supported provisional restoration.

During the dental examination, all maxillary teeth appeared with reduced periodontal support, gingival recessions and poor prognosis due to extended bone resorption and attachment loss [12] (Figure 1A). The mandibular teeth had received periodontal therapy with a good response, and the patient followed regular recalls. The patient desired prosthodontic treatment for his maxilla, as he could chew and perform music only with great insecurity, due to the situation of the maxillary teeth.

The radiographic examination with a panoramic X-ray and a CBCT confirmed the clinical findings (Figure 1B). Extensive pneumatization of the sinuses bilaterally and generalized marginal bone loss were observed.

The patient expressed his desire for a fixed restoration in the maxilla to avoid a removable complete denture. He also denied a sinus lift because he was afraid that he would not be able to work and function properly for some time. He also insisted that he would not remain without a fixed restoration even for a single day. The treatment plan, as agreed with the patient, included a presurgical diagnostic phase; the fabrication of a tooth- and tissue-supported provisional restoration; the insertion of six implants (if possible, with immediate loading); the fabrication of an implant-supported temporary restoration, and the final implant-supported FDP. All clinical data were selected and handled according to the Declaration of Helsinki, and the patient signed an informed consent form permitting the communication and publication of clinical images in scientific forums.

In the first phase, an initial diagnostic digital wax-up was performed, and a provisional teeth-and-tissue-supported FDP was planned, which would be retained by the maxillary canines and supported by the retromolar areas. This “tripodic” (tooth-and-tissue-supported provisional restoration is described in the literature as an alternative fixed prosthesis to avoid a complete denture during the bone healing and implant osseointegration phases [13].

Teeth #13 and #23 were treated endodontically, and a core build-up was performed with fiber posts and composite material. Teeth #14, #21, #24, #25, and #27 were extracted, and Guided Bone Regeneration was performed with bone grafting material (4Bone BCH, MIS Co., Israel) and a resorbable collagen membrane (4Bone RCM, MIS Co., Israel) in the 2nd quadrant. The provisional “tripodic” restoration was cemented on maxillary canines with temporary cement (Temp-Bond, Kerr Co., CA, USA). A major advantage of this provisional restoration compared to a removable prosthesis is the avoidance of pressure on grafted areas (Figure 1C,D) for the healing period, as well as the patient’s comfort due to the absence of palatal coverage.

The digital implant planning was performed using a special software (MSoft, MIS Co., Israel). A surgical guide was 3D printed for guided implant placement in the maxillary regions: 15, #13, #11, #21, #23, and #25. A new provisional implant-retained restoration was designed using the same software and was fabricated by 3D printing, planned to be delivered on multi-unit transmucosal abutments at the time of implant insertion.

**Figure 2:**
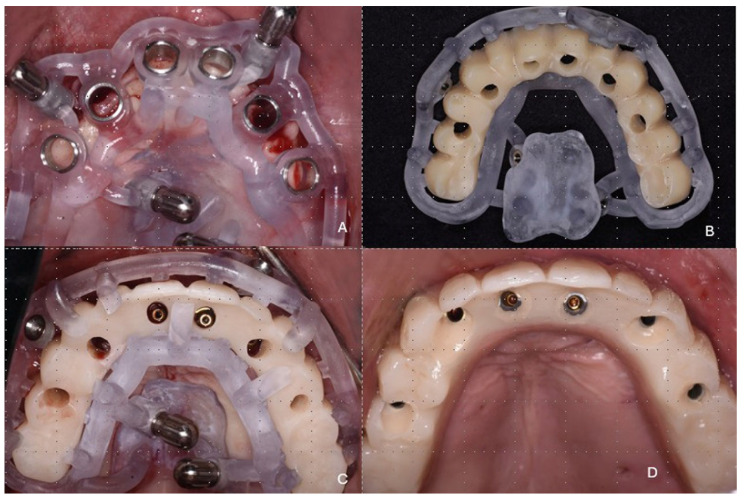
(**A**) The surgical guide fixed with anchoring pins, (**B**) The guide for the placement of the implant-supported provisional restoration, (**C**) The provisional restoration over the temporary copings on the multi-unit abutments, (**D**) The provisional restoration fixed on the abutments.

During implant surgery, canines were extracted, and the surgical guide was stabilized on the edentulous maxilla with three buccal and two palatal stabilization pins. For the precise positioning of the provisional restoration over the implants, a second guide was fabricated that could be stabilized with the same anchoring pins at the same bone sites (Figure 2A,B).

Dental implants with conical connection (MIS C1 implants 4.20/10 mm, MIS Co., Israel) were placed fully guided transmucosally without raising a flap in the selected regions #15, #11, #21, #25, as well as in the post-extraction sites #13, #23. Sufficient initial stability (>40 Ncm torque) was achieved for all implants. Straight transmucosal multi-unit abutments (multi-unit abutments, MIS Co., Israel) of appropriate gingival height (GH) were selected intraoperatively, according to the soft tissue thickness, and were fixed on the implants with the torque recommended by the manufacturer. Immediate loading was performed with the pre-fabricated screw-retained PMMA provisional restoration attached to titanium copings (temporary cylinders, MIS Co., Israel) over the transmucosal abutments with auto-polymerizing acrylic resin.

The implant-supported provisional restoration was aligned over the implants and the corresponding multi-units using the second guide (Figure 2C,D), which allowed precise orientation of the prosthesis according to the planned design.

**Figure 3:**
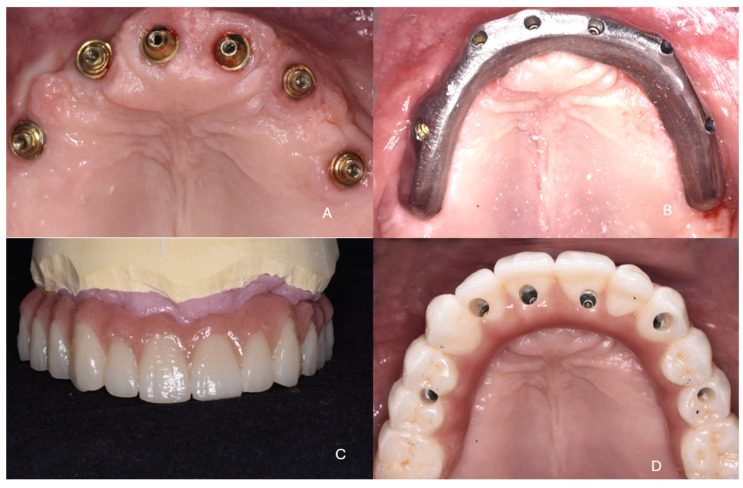
(**A**) Clinical situation at the end of osseointegration, (**B**) The Ti bar over the implant abutments, (**C**,**D**) The final restoration of monolithic zirconia on Ti-bar.

The osseointegration period was uneventful (Figure 3A), and four months later, a final digital scan was made. The STL files were mounted in the digital articulator with the existing provisional as guide points. A diagnostic digital wax-up of the final restoration was performed, hence integrating the alterations on the occlusal surfaces that occurred during this time. A verification splint fabricated from PMMA incorporating impression posts was used to check the accuracy of the impression. A Ti-bar (titanium-bar) was designed and fabricated to passively fit over the six multi-unit abutments (Figure 3B). This was followed by fitting a prototype restoration over the Ti-bar, to check esthetic details and occlusal contacts.

Over the Ti-bar, a milled monolithic zirconia restoration (Prime, Ivoclar, Schaan, Lichtenstein) was fabricated, restoring the teeth and the missing soft tissues. After the final try-in, the zirconia restoration was returned to the lab and was cemented on the Ti-bar after appropriate conditioning of the surfaces with a dual-polymerization resin cement (Panavia, Curaray Co., Tokyo, Japan) (Figure 3C,D).

**Figure 4:**
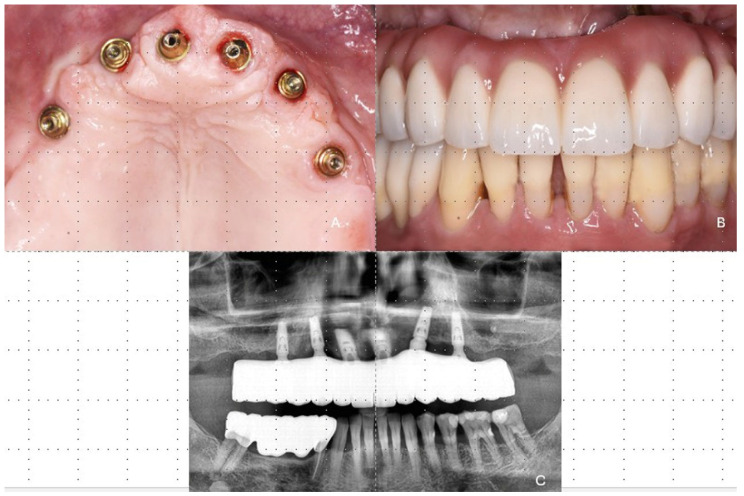
(**A**,**B**) Clinical situation at 1-year recall, (**C**) Radiographic examination at 1-year recall.

At the 1st year recall, a stable condition was confirmed clinically and radiographically, and no marginal bone loss was detected (Figure 4A–C). The patient’s functional and esthetic demands were successfully met with the described treatment, as subjectively based on the patient’s perception and reported comments.

**Case 2**

**Figure 5:**
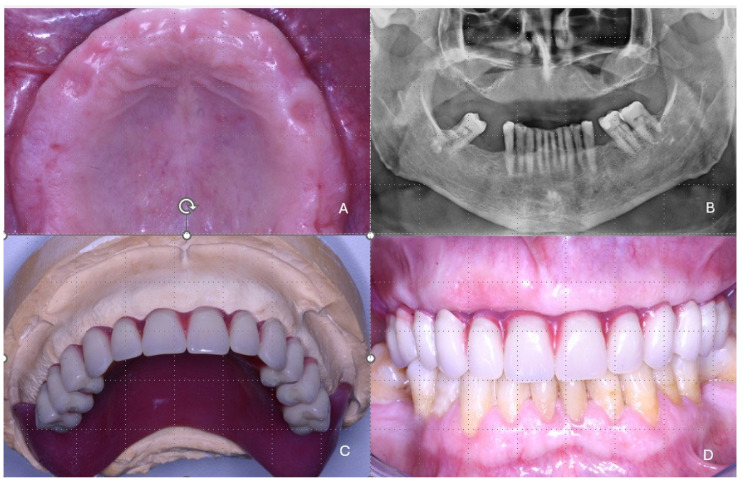
(**A**) Initial clinical situation, (**B**) Initial radiographic situation, (**C**) Diagnostic set-up, (**D**) Try-in of the diagnostic set-up to the patient.

A 67-year-old male Caucasian patient presented for treatment for his completely edentulous maxilla in a private clinic (Figure 5A). No significant medications or disease were reported in medical history. The remaining maxillary teeth had been extracted several years ago for periodontal reasons, and the patient was not satisfied with the provided immediate complete denture. For this reason, he expressed the desire for a fixed restoration as soon as possible. The mandibular teeth had undergone periodontal treatment, and the patient followed a regular recall program.

A treatment plan for an implant-supported fixed full-arch restoration was decided with the patient for restoring the maxillary arch. Under the condition of adequate primary stability of the implants, immediate loading with a provisional restoration would be performed. The available bone height in the maxillary molar area was not adequate for implant placement, and the patient refused any surgical procedure in the sinuses (Figure 5B). For this reason, an all-on-four implant-supported FDP was planned for the maxilla.

A conventional set-up was made on a base plate without a labial flange and was checked intraorally (Figure 5C,D). The lip support offered by the teeth was considered adequate; there was no need for a labial flange. The decision for a fixed restoration was confirmed, and it was decided that an FP-2 or FP-3 prosthesis would be fabricated. The implant insertion was planned digitally based on the teeth set-up, which was also digitized by a laboratory scanner.

**Figure 6:**
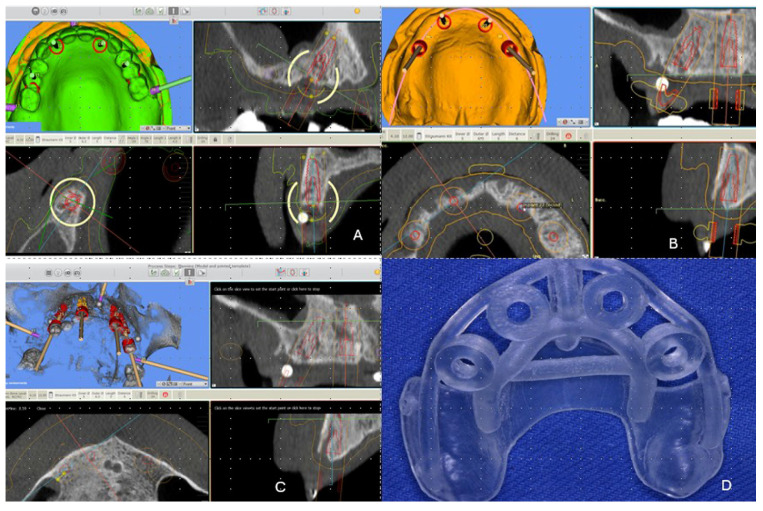
(**A**,**B**) Presurgical digital planning by superimposition of the STL files of the initial cast, the wax-up, and the CBCT. (**C**) Digital design of the surgical guide, (**D**) The printed surgical guide.

The STL files from the master cast, the set-up and the CBCT were superimposed using the SMOP software (SMOP, Swissmeda Co., Baar, Switzerland) (Figure 6A–C). A surgical guide was designed that could be fixed by three anchoring screws, leaving adequate space for flap elevation (Figure 6D).

**Figure 7:**
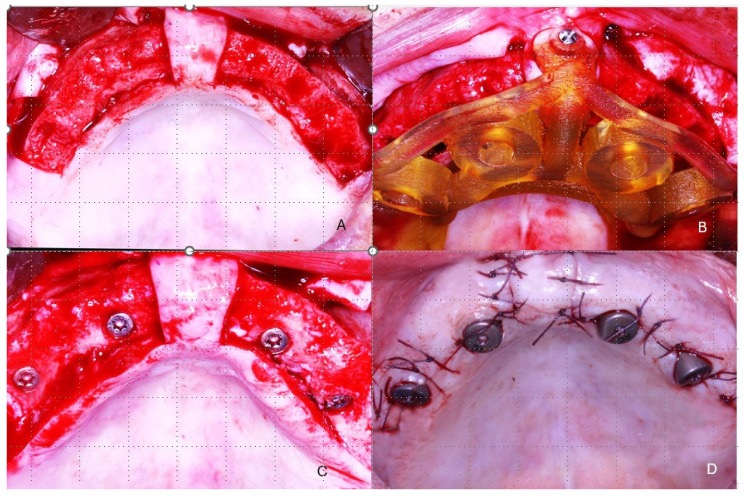
(**A**) Elevation of mucoperiostal flaps, (**B**) Fixation of the surgical guide after flap elevation, (**C**) Insertion of four implants, (**D**) Suturing around healing abutments.

The 3D-printed surgical guide was fitted to the patient, drilling of the anchoring sites was performed, and subsequently it was removed for the flap elevation to follow. Two separate full-thickness mucoperiostal flaps were elevated on both quadrants, leaving in the midline an intact zone of mucosa to ensure the correct seating of the surgical guide. Four implants (Straumann BLT RC 4.1 mm × 12 mm, Straumann Co., Basel, Switzerland) were inserted with a fully guided procedure as planned (Figure 7A–C). The distal implants were intentionally inserted with a distal inclination to avoid the sinus floor and increase the anterio-posterior spread. The flaps were sutured around the healing abutments (Figure 7D).

**Figure 8:**
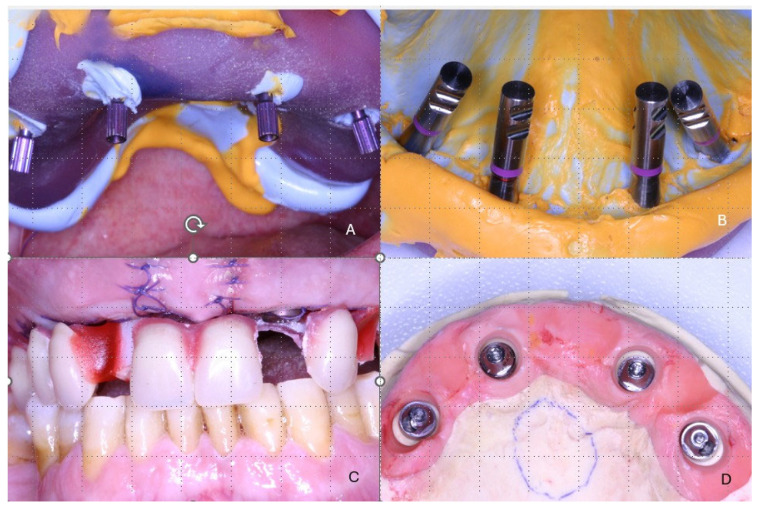
(**A**) Conventional open tray impression, (**B**) Fitting of implant analogs, (**C**) Fitting of the teeth set-up over the implants and indexing with auto-polymerizing resin on healing abutments, (**D**) The working cast with selected multi-unit abutments.

All four implants had adequate initial stability (>40 Ncm torque), and immediate loading was decided. After surgical procedure completion, impression posts were fixed, and a conventional implant-level impression was made using a custom open tray and addition-type silicone (Figure 8A,B). For registration of the interarch relation, the initial diagnostic set-up was modified to fit over the healing abutments. After indexing them with an auto-polymerizing resin, it was sent to the laboratory along with healing abutments identical to the ones used intraorally (Figure 8C).

A conventional stone master cast was fabricated and mounted in the articulator using the modified set-up and the bite registration. Multi-unit abutments were selected (Figure 8D) according to the soft tissue thickness (Straumann SRA abutments, Straumann Co., Basel, Switzerland) measured in the gingival tissue silicone mask (Figure 8D).

**Figure 9:**
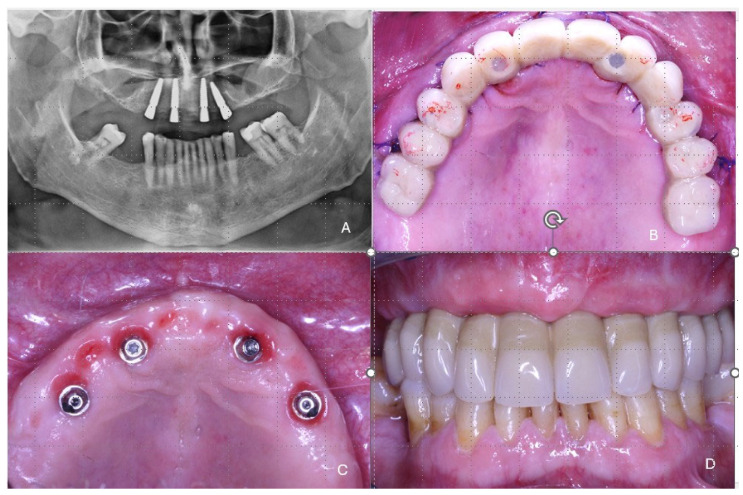
(**A**) Radiographic examination after implant insertion, (**B**) The provisional restoration, (**C**,**D**) Clinical situation at the end of osseointegration.

A radiographic examination was performed prior to the fabrication of the final restoration to verify the successful placement of the implants (Figure 9A). A screw-retained metal-acrylic FDP was fabricated using plastic castable copings for SRA abutments. The cervical part of the restoration was formed mimicking the root, due to the resorption of the alveolar ridge. 48 h later, the selected multi-unit abutments were fitted onto the implants with the recommended torque (35 Ncm), followed by prosthesis delivery with prosthetic screws torqued at 15 Ncm and occlusal adjustment, to ensure a mutual protection scheme (Figure 9B). The osseointegration period was uneventful, and four months later, the clinical situation was stable (Figure 9C,D).

## 2. Discussion

The two cases presented in this report have a different initial clinical situation, one with terminal maxillary dentition and the other with complete edentulism, yet both resulted in a fixed prosthesis. The treatment plan for both cases was an implant-supported FDP in the maxilla, including fully guided and prosthetically driven implant placement with immediate loading. Important milestones to consider along these different treatment pathways include the collection of preliminary clinical and radiographic data, digital planning of implant placement, intermaxillary relation recording, and prosthesis fabrication for immediate loading.

The first step in guided implantation is detailed pre-surgical planning based on radiographic evaluation and digitalization of the jaw that will be restored. Intraoral scanning was performed in Case 1, but in the edentulous Case 2, a conventional impression was made, producing a stone cast that was subsequently scanned in the dental laboratory. A CBCT provides a 3D image of the bone substrate, teeth, and surrounding structures, so that the surgeon to view the patient’s anatomy from multiple angles and assess the quality and quantity of the available bone.

CBCT data in the DICOM (Digital Imaging and Communication in Medicine) format, which is the standard for medical imaging, is imported into the design software, followed by the superimposition of the STL files (Standard Tesselation Language) that describe three-dimensional intraoral maxillary anatomy [6]. The superimposition of the diagnostic wax-up can also be used to provide better guidance for optimal implant position in relation to the final prosthesis. Implant planning software allows virtual placement of each implant in the ideal prosthetic position. Parameters such as implant diameter, length, and angulation can be determined at this stage. Additionally, the software allows the surgeon to assess the proximity of the implant to critical structures like the maxillary sinus, inferior alveolar nerve, and adjacent teeth [14]. A fundamental factor for the accuracy of the treatment plan is the precise matching and superimposition of the intraoral scan with the CBCT, which must be thoroughly controlled by the clinician [15]. In the first case, the remaining maxillary teeth provided multiple reference points for accurate, definitive stacking of relative files, whereas in the second case of complete edentulism, various protocols have been suggested depending on the planning software used. Dual scanning, intraoral application of resin markers, and temporary implants are used to allow solid overlay of STL files on DICOM files [16,17]. The planning software used in the second case utilizes the surface outline of vertical sections to confirm the correct matching of CBCT and surface scan, simplifying the clinical process [18]. Most design softwares have adequate accuracy to allow estimation of the soft tissue thickness and predict the height of the appropriate transmucosal abutment, which can be inserted immediately after implant placement [19].

An important stage in the treatment plan procedure for fixed implant restoration of the edentulous maxilla is the evaluation of lip support [20]. Extensive bone resorption may require an anterior flange or non-ideal inclination of artificial teeth, rendering a removable prosthesis the better restorative option. The second case presented an alternative way to evaluate the tooth set-up without any anterior flange, allowing at the same time the assessment of the tooth-ridge vertical and horizontal distance that can provide further information regarding the prosthesis design. When necessary, a provisional restoration can be designed and fabricated before implant placement, based on the presurgical plan. Thus, in cases where sufficient primary stability of the implants can be achieved, immediate loading of the implants can be performed. The wax-up of the planned restoration can be an analog wax-up scanned with a laboratory scanner, or a purely digital wax-up created directly in design software [14].

Once the presurgical plan is complete, a surgical guide is fabricated using 3D printing or milling technology. This guide is customized to fit in the patient’s mouth and includes openings corresponding to the planned implantation sites. Moreover, surgical guides can be tooth-supported, mucosa-supported (for edentulous patients), or bone-supported, depending on the clinical characteristics of each case, ensuring that the implant is placed exactly as planned in the pre-surgical design [2]. This versatility allows guided implantation across a wide variety of cases, from single-tooth implants to full-arch reconstructions [6]. The distribution of the teeth chosen to stabilize the guide should be as symmetrical as possible, and teeth with increased mobility should be avoided [21]. A systematic review of the precision of surgical guides showed greater accuracy for tooth-supported compared to bone-supported guides [22]. Another systematic review on the accuracy of digitally designed and fabricated surgical guides concluded that bilateral tooth-supported guides demonstrated the highest accuracy in vitro and similar accuracy in vivo with unilateral tooth-supported guides. Mucosa-supported guides exhibited the lowest accuracy in vivo. The design of supporting elements, fixation screws, and sleeves of implant guides can affect the accuracy of guided implant surgery [23].

The stability of the surgical guide can be increased by anchor pins designed in areas distant from the implant sites [24]. Usually, three to five anchor pins are used in the edentulous maxilla and three to four in the edentulous mandible. In the cases presented in this report, palatal extension of the surgical guide in the first case and tripodic stabilization on mucosal seating in the second case secured the precise drilling and engagement of stabilizing anchors. Although teeth can guide the correct seating and orientation of a surgical guide before their subsequent extraction, in our first case, this was not applicable due to the immediate implantation at canine sites. The use of fixation pins increased the accuracy of implant placement in an in vitro study [25]. Similar results were reported for the distal free end situation [26]. On the other hand, only minimal differences were noted in an in vitro study comparing guided implant placement in a free-end situation with and without fixation pins on the surgical guides [25]. Regarding edentulous patients, a systematic review on the accuracy of tissue-supported guides with pin fixation concluded that tissue-supported guides can be a reliable and predictable treatment option for implant restorations [27].

The accuracy in the fabrication of surgical guides is an important factor that might influence the overall accuracy of the surgical procedure. An in vitro study on the accuracy of a broad range of 3D printers showed that the current generation of 3D printers could produce clinically acceptable levels of accuracy [28]. In another in vitro study, no significant differences in any surgical accuracy indicators between additive and subtractive manufactured guides were noted [29].

During surgery, the surgical guide determines the angulation, depth, and position of the drill, ensuring that the implant is inserted accurately at the planned position. This precision reduces the risk of errors and complications such as improper alignment, perforation of the sinus or nerve, and uneven loading of the implants [30]. In a recent clinical study, lower deviation was noted for guided implant placement, ranging from 0.97  ±  0.37 mm at the cervical and 1.13  ±  0.36 mm at the apical implant position. The difference in angle deviation between the planned implant and the placed implant was 3.42  ±  2.12° [31]. Taking into consideration the aforementioned risk of deviation, a safety margin of 2 mm from adjacent anatomical structures is the accepted standard for presurgical planning [32].

The guide sleeves can be either open or closed. Open sleeves with a C-shaped buccal opening are the optimal choice for posterior areas where mouth opening and inter-arch space are limited or insufficient. A smaller distance from the sleeve to the bone leads to more accurate results [33]. The sleeve design also affects the accuracy of the surgical guide [34]. Sleeve heights of ≤5 mm inevitably result in deviation of implant placement and a notable reduction in accuracy [35].

Another factor affecting implant accuracy is the sleeve-bone distance. With a sleeve-bone distance of 2 or 4 mm, the accuracy of closed and open sleeves is similar. However, at a sleeve-bone distance of 6 mm, both open and closed sleeves show lower accuracy, with open sleeves exhibiting a more significant trend [36]. Both cases presented herein utilized printed surgical guides that accepted handheld metal sleeves for drill-guidance of increased accuracy. Metal sleeves have shown greater accuracy compared to sleeveless guides as they provide more guidance and stability during drilling. However, factors such as drilling depth, sleeve design, and bone density should be carefully considered to optimize accuracy [37,38]. Although guided surgery safeguards the procedure and ensures the correct 3D implant position, it is also combined with increased costs for guide production and a learning curve for the successful clinical application [39]. Fundamental anatomic knowledge, an interrelated understanding of surgical and prosthetic parameters, and advanced surgical skills are essential prerequisites for the supporting integration of guided surgery in contemporary therapeutic procedures.

Despite those issues, based on the results of a systematic review, implant failure rates were almost three times higher in freehand placement than in fully guided placement [40]. Another advantage of guided implantation is that, in many cases, it can be performed as a minimally invasive procedure with small or no incisions and minimal trauma to the surrounding tissues. This leads to faster healing times, reduced postoperative discomfort, and better patient outcomes [41]. Moreover, flapless surgery has been shown to be more accurate compared to free-hand [42]. On the other side, a systematic review on minimal invasive implant placement showed that even with detailed planning, deviations from the originally planned position may still occur, and in 7% of the included cases, the implants could not be placed with the flapless protocol [41]. In the second case, planning was performed without any fixed, definitive reference points, and possible inaccuracies may have developed. For this reason, a flap was elevated to permit visual inspection during surgery. Deviation of implant positions affects not only the relationship to anatomical structures but also the provisional prosthesis, if immediate loading is planned [43]. The first case presented a fully guided protocol, in which a provisional prosthesis was related to the implants with a prosthetic guide engaging with the same fixation pins. Intraoral attachment to temporary cylinders with self-polymerizing resin allowed passivity despite possible placement deviations.

Immediate loading after guided surgery was mentioned several years ago in a case-series study with 10 patients, reporting a survival rate of 100% [44].

Another case series on 12 patients reported a survival rate of 98.2% at one-year recall [45]. Immediate loading of implants with temporary restorations was reported several years ago, but the first publications were on single implants, mainly in the anterior area [46,47,48]. A case report also recently described immediate restoration by means of guided surgery for a single implant [49]. Other case reports following the same protocol as the present study have also been published, demonstrating similar positive results [50,51].

A pilot study with 55 implants in 10 patients with a similar protocol as in the present study reported a survival rate of 98.18% (one implant failed) without prosthetic complications and minimal marginal bone loss [52].

In another case series of 10 patients, in thirteen arches, implants were loaded immediately after teeth extractions and implant placement with full-arch screw-retained resin transitional prostheses. The clinical series resulted in 100% clinical implant and prosthetic survival rates [53]. Immediate loading can be performed more commonly in a time frame of 0–72 h [54], but a period of up to one week has also been reported [55]. In the second case described in this report, an implant impression was performed intraoperatively, and prosthesis fabrication was initiated after surgery. Although immediate loading is performed with a provisional prosthesis, in the present case, the final prosthesis for immediate loading was completed and delivered in 48 h. The prosthesis was a metal-polymer restoration, allowing future mucosal modification after tissue maturation to the final morphology.

Immediate loading of implants in edentulous patients was reported in a clinical study including 22 patients with 8–10 implants in the mandible. The survival rate in the follow-up period (84.2 ± 4.9 months) was 97.5% of the implants, and the rate of prosthetic complications was 27.3%. The immediate provisionalization was not associated with the clinical outcomes of the study, according to the authors [56]. Another clinical study on 32 patients and 285 implants employed the same protocol as in the present study (guided implant surgery and double guide). In a follow-up period of three years, the cumulative implant survival rate was 97.54%, and the marginal bone loss was 1.32 ± 0.41 mm [3]. Fully edentulous cases planned for immediate loading present the challenge of accurate centric relation and vertical dimension recording for precise cast mounting (physical or virtual), a process that may extend or delay the clinical procedures [57]. In the first case, a completely digital workflow allowed the correct virtual cast mounting and the completion of all auxiliary and prosthetic procedures before any surgery took place. In the second case, however, the preoperative teeth setup on the modified base plate was utilized intraoperatively to register the selected implant positions and allow, through its stable relationship with the master cast, the correct mounting of the latter without time delay or an additional appointment. In a recent study [58], an impression reference technique has been presented, where digital and traditional methods are combined for the correct inter-arch relationship recording of edentulous jaws undergoing immediate loading.

Summaries of clinical outcomes of guided and minimally invasive surgery have been reported in recent systematic reviews with encouraging results [6,41]. Immediate loading and guided implant placement also show clinical advantages for elderly patients, who do not wish repeated surgical procedures, and their efficacy has been shown [46,47]. The dilemma, however, of retaining or extracting a tooth with a questionable prognosis remains for the clinician, who should take into consideration the characteristics of each clinical case [48].

Guided implant surgery and immediate loading are technique-sensitive and demanding procedures that include several factors that may affect the final outcome negatively. These factors include the CBCT quality, the accuracy of DICOM and STL digital files and their superimposition, the possible deviation from the original planning, the precision of fabrication, the fit of the provisional restoration, the increased cost, and the needed learning curve from the clinician.

This clinical report of two cases focused on fully guided implant placement for full-arch maxillary restorations and immediate loading with screw-retained FDPs, confirming that analog and digital workflows can be used exclusively or in combination for achieve successful treatment. Although the clinical outcome is encouraging, it is a case report of two patients with successful repeatability and standardization of the procedure, which remains to be confirmed by a larger-scale study.

## 3. Conclusions

Within the limitations of these cases’ presentation, it can be assumed that guided implant surgery and immediate loading with a fixed restoration are viable restorative options for the treatment of the edentulous maxilla opposing a dentate mandible. More clinical trials are needed to verify the long-term success of the presented clinical protocol.

## Data Availability

The data presented in this study are unavailable on request from the corresponding author due to privacy and ethical restrictions.
